# Mitral Annular Calcification and Thromboembolic Risk

**DOI:** 10.3390/life13071568

**Published:** 2023-07-15

**Authors:** Paula Cristina Morariu, Daniela Maria Tanase, Diana Elena Iov, Oana Sîrbu, Alexandru Florinel Oancea, Cornel Gabriel Mircea, Cristina Petronela Chiriac, Genoveva Livia Baroi, Ionela-Daniela Morariu, Cristina Gena Dascălu, Laurenţiu Şorodoc, Mariana Floria

**Affiliations:** 1 Department of Internal Medicine, University of Medicine and Pharmacy “Grigore T. Popa”, 16 University Street, 700115 Iasi, Romania; adascalitei.paula-cristina@d.umfiasi.ro (P.C.M.); dr.oana.sirbu@gmail.com (O.S.); oancea.alexandru-florinel@d.email.umfiasi.ro (A.F.O.); mircea.cg@gmail.com (C.G.M.); livia.baroi@umfiasi.ro (G.L.B.); laurentiu.sorodoc@umfiasi.ro (L.Ş.); floria.mariana@umfiasi.ro (M.F.); 2 Medical Clinic, “Sf. Spiridon” Emergency Hospital, 700111 Iasi, Romania; chiriac.cristina@spitalspiridon.ro; 3 Cardiology Clinic, “Sf. Spiridon” Emergency Hospital Iași, 700111 Iasi, Romania; 4 Surgery Clinic, ”Sf. Spiridon” Emergency Hospital Iași, 700111 Iasi, Romania; 5 Department of Environmental and Food Chemistry, “Grigore T. Popa” University of Medicine and Pharmacy Iași, 700111 Iasi, Romania; ionela.morariu@umfiasi.ro; 6 Department of Medical Informatics and Biostatistics, Faculty of Dental Medicine, University of Medicine and Pharmacy “Grigore T. Popa”, 16 University Street, 700115 Iasi, Romania

**Keywords:** mitral annulus calcification, thromboembolic risk, stroke, CHA_2_-DS_2_-VASc score, atherosclerosis

## Abstract

Thromboembolic (TE) risk scores used for atrial fibrillation (AF) patients do not include mitral annular calcification (MAC) as a potential indicator of vascular disease. This research evaluated the correlation between MAC and TE risk scores (CHADS_2_ and CHA_2_DS_2_-VASc). We compared TE risk score values and clinical and echocardiographic data in patients with and without MAC. We included, prospectively, 103 patients: 40.8% with AF, 83.5% with hypertension, 30.1% with type II diabetes mellitus, 79.6% with chronic heart failure, and 7.8% with a history of stroke. We identified MAC in 50.5% of patients. The mean CHADS_2_ and CHA_2_DS_2_-VASc scores were 2.56 ± 1.135 and 4.57 ± 1.61, respectively. In MAC patients, both scores tended to increase significantly compared with the control (2.88 ± 1.114 versus 2.24 ± 1.06, *p* = 0.005, and 5.21 ± 1.51 versus 3.92 ± 1.46, *p* < 0.001, respectively). The left ventricular ejection fraction negatively correlated with the presence of MAC (r = −0.254, *p* = 0.01). The presence of MAC was a risk factor for vascular disease (OR = 2.47, χ^2^ = 34.32, *p* < 0001). Conclusions: The presence of MAC is associated with greater TE risk scores and a higher risk of vascular disease. It appears that adding MAC as a vascular disease parameter to TE risk scores may have benefits for patients by improving their predictive value.

## 1. Introduction

Before becoming symptomatic and affecting patient survival, atherothrombosis and valvular calcifications involve a silent process. The early identification of the clinical significance of these calcifications is essential for the appropriate classification of patients into TE risk groups in order to produce practical prevention methods for adverse cardiac events.

Mitral annular calcification (MAC) is the result of a chronic, degenerative, and progressive process of calcification of the fibrous mitral annulus [1]. Although MAC was first thought to be an age-associated process, current research shows that it is an active mechanism that involves inflammation, hemodynamic stress, lipid deposition, and the production of new bone [2]. In many cases, it is discovered alongside other atherosclerotic risk factors such as tobacco use, arterial hypertension (HTN), obesity, dyslipidemia, and type II diabetes mellitus (DM) [3].

Despite being an incidental finding and frequently asymptomatic, MAC has clinical relevance because of its correlation with a greater number of cardiovascular diseases, which independently affect cardiovascular and all-cause mortality. It is also related to mitral valve disease and conduction abnormalities [2,4,5]. Recent research has found a link between MAC and the risk of stroke. In patients without clinical cardiovascular disease, MAC serves as an independent predictor of stroke [6,7].

The CHA_2_DS_2_-VASc score and its predecessor, CHADS_2_, are used to predict TE risk in patients with AF [8]. An elevated risk of TE events is attributed to vascular involvement (history of myocardial infarction—MI, aortic atherosclerosis plaques, peripheral arterial disease—PAD), but further research is needed to determine the role of MAC. The current data attest to a correlation between MAC and TE risk to some level. To the best of our knowledge, the clinical importance of MAC in a patient group has not yet been fully characterized; however, data from the literature reveals relationships that are statistically significant and may have therapeutic implications.

The aim of this study was to evaluate the postulated usefulness of MAC as a possible marker of vascular disease for TE risk calculation, in addition to other parameters of the CHA_2_DS_2_-VASc and CHADS_2_ scores.

## 2. Materials and Methods

### 2.1. Patient Enrolment, Inclusion, and Exclusion Criteria

This study is a pilot, retrospective study carried out in the Internal Medicine Clinic of the “Sfântul Spiridon” Emergency Hospital, Iași, Romania.

Patients with or without calcification of the mitral annulus who were over the age of 18 with the ability to comprehend and accept an informed consent form were included in this research. The other inclusion criteria were the diagnosis of HTN, dyslipidemia, type II DM, ischemic heart disease (IHD), angina pectoralis, or chronic MI.

Patients under the age of 18 years; patients without signed informed consent; patients with chronic kidney disease with creatinine clearance below 30 mL/min/1.73 m^2^, hemodynamically significant valv diseases (more than mild severity), mechanical prostheses, or aortic or mitral valve reconstruction; and patients with surgical myocardial revascularization were excluded from the study.

### 2.2. Clinical Investigation and Data Collection

For the patients who were enrolled, we gathered clinical, biochemical, electrocardiographic (ECG), and echocardiographic data. Thromboembolic risk was determined using the CHADS2 and CHA2DS2-VASc scores. These variables were compared in patients with MAC (study group) and those without MAC (control group).

Sex, age, body surface area (BSA), and body mass index (BMI) were included in the demographic characteristics. The patients’ smoking or non-smoking status was also assessed. We also noted patient-associated comorbidities such as a history of HTN, heart failure (HF), DM, aortic atherosclerosis plaques, dyslipidemia, peripheral arterial disease (PAD), IHD, myocardial infarction (MI), transient ischemic attack (TIA), and stroke. Chronic treatment with statins, oral anticoagulants, or antiplatelet agents in all patients was noted.

For the patients’ biological profiles, we gathered information on their total cholesterol and triglyceride levels and conducted liver function tests, including alanine aspartate transferase (AST), alanine aminotransferase (ALT), gamma-glutamyl-transferase (GGT), and fasting blood sugar levels.

The CHADS_2_ score assigns 1 point each for congestive HF or a left ventricular ejection fraction (LVEF) below 40% (C), HTN (H), age over 75 years (A), and DM (D) and 2 points each for a previous stroke and TIA or systemic thromboembolism, totaling a maximum of 6 points. The CHA_2_DS_2_-VASc score differs from the first by adding other parameters—vascular disease (history of MI, PAD, or the presence of atherosclerotic plaques on the aortic cross) for which 1 point is given; female sex, 1 point; age between 64 and 75 years, 1 point; and over 75 years, 2 points, with a maximum value of 9 points [9]. In addition, recently, hypertrophic cardiomyopathy was added to the letter C in the acronym.

All patients underwent an ECG to determine the presence of sinus rhythm or AF upon admission, before transthoracic echocardiography.

**Figure 1:**
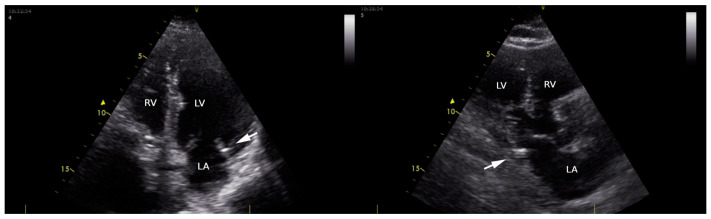
Transthoracic echocardiography in apical four-chamber view (**left**) and long parasternal view (**right**), showing mitral annular calcification (arrow) in an enrolled patient. LA: left atrial; LV: left ventricle; RV: right ventricle.

A cardiac structural and functional assessment, including the presence of MAC, was conducted via transthoracic echocardiography using a Vivid T8 Pro, GE Healthcare (according to the newest recommendations of the European Association of Cardiovascular Imaging) [10]. MAC (Figure 1) was defined as an echo-dense structure, located at the junction of the atrioventricular groove and the posterior or anterior mitral leaflet on the parasternal long-axis view; apical 4- or 2-chamber view; and parasternal short-axis view [11]. The parameters quantified during the transthoracic echocardiographic examination were as follows: left ventricular (LV) dimensions (interventricular septum—IVS, left ventricular posterior wall—LVPW, left ventricular end-diastolic diameter—LVEDD); LV systolic function (LV ejection fraction via Simpson method—LVEF), and LV diastolic function (E/A; E/e’; left atrium volume). E/A ratio was assessed in patients in sinus rhythm in apical 4- or 2-chamber view; E represents the maximum velocity of early diastolic filling of the mitral flow, and A represents the maximum velocity of the late diastolic filling of mitral flow. The diastolic index or E/e’ ratio (the ratio of early mitral in-flow to tissue velocity of the mitral annulus) was assessed in apical 4- or 2-chamber view in patients with AF (A wave being absent in these patients). We used the e’ velocity obtained via Tissue Doppler Imaging from the septal and lateral mitral annulus. For patients in AF during the echocardiographic examination, we used average velocity measurements taken during 10 consecutive cycles.

### 2.3. Statistical Analysis

Statistical analysis was performed using the SPSS version 29.0 software package (SPSS Inc., Chicago, IL, USA). Categorical variables were presented as frequencies and percentages and continuous variables were presented as mean ± standard deviation. Categorical and ordinal variables were compared between groups using the χ^2^ test, and continuous variables were compared between groups using Student’s *t*-test and the Mann–Whitney test (when the precondition of normal repartition of values was not verified). Pearson and Spearman correlation coefficients were used to assess the degree of association between variables (Pearson for continuous variables and Spearman for nominal or ordinal variables), and binary logistic regression was used for multivariate analysis. The association between continuous variables, if any, was analyzed through linear regression. Statistical significance was assessed at a value of *p* < 0.05, and the confidence interval (CI) was 95%.

## 3. Results

A total of 103 patients satisfied the requirements of the study, and they were divided into two groups: those with MAC present at the time of the echocardiogram examination (52 patients), and those without MAC present at the time of the echocardiography assessment (51 patients). To fulfill the outlined goals, a variety of comparisons and statistical assessments were performed between the two groups.

### 3.1. Study Group Description

In this retrospective study, we enrolled 103 patients, of whom 57 were women (55.3%) and 46 were males (44.7%). The mean age was 72.59 years ± 9.9 years. The general characteristics of the patients included are summarized in Table 1. The data are presented in the table both for all patients and grouped according to the presence or absence of MAC.

An echocardiographic assessment of the LV systolic function showed a mean LVEF of 49.50%, with a standard deviation of 13.085%, a minimum value of 10%, and a maximum value of 74%. Other mean values calculated based on echocardiographic data were as follows:

- Interventricular septum (IVS): 12.14 ± 1.754 mm;
- LV posterior wall: 12.16 ± 1.685 mm;
- LV end-diastolic diameter (LVEDD): 50.82 ± 9.045 mm.

### 3.2. Relationship between MAC and Clinical, Biological, and Echocardiographic Parameters

Patients with MAC had a significantly greater prevalence of DM, aortic atherosclerosis plaques, and vascular disease. When compared with individuals without MAC, they were also significantly older.

According to the laboratory tests, individuals with MAC had non-significantly smaller triglyceride levels than those without MAC (114.94 ± 59.352 vs. 120.61 ± 47.808 mg/dL); in the case of hepatic function, non-significant variations were registered; patients with MAC presented non-significantly increased fasting glycemia compared with the patients without MAC (126.85 ± 61.046 vs. 113.78 ± 26.418 mg/dL).

When compared with the control group, the study group’s LV ejection percent was considerably lower (46.48 ± 13.126% vs. 52.59 ± 12.423%). In addition, sinus rhythm patients (62.83%) with MAC showed a significantly decreased LVEF as compared with those without MAC—55.73 ± 12.3% vs. 46.96 ± 14.5%, *p* = 0.013. The difference in LVEFs between MAC patients and the control was not significant in the AF patients (45.92 ± 11.594% vs. 46.83 ± 10.601%, *p* = 0.794).

### 3.3. Relationship between MAC and Other Comorbidities

We also emphasized the association between MAC and other diseases in this investigation. When compared with the control group, the patients from our research with MAC often had a greater prevalence of DM (*p* = 0.006). Among the 52 patients who had MAC, 22 of them (42.3%) also had DM, and among the 51 patients without MAC, only 9 patients (17.6%) had DM, values which indicate DM as a risk factor for MAC (OR = 3.422).

Only 19 patients (37.3%) of the 51 patients without MAC had vascular disease, compared with 48 (92.3%) of the 52 patients with MAC who also had vascular involvement. Therefore, the presence of vascular disease was a risk factor for MAC in our research (OR = 20.211). The presence of aortic atherosclerosis plaques is also significantly associated with MAC: 47 patients with MAC had this diagnosis (90.4%), compared with only 21 patients without MAC (41.2%), leading to a calculated risk of OR = 13.429.

Regarding the other investigated comorbidities, no significant associations with MAC were identified, even though among the patients with MAC we found increased incidences of HTN (88.5%), obesity (38.5%), prior stroke (11.5%), AF (46.2%), and PAD (23.1%).

### 3.4. Multivariate Analysis of the Relationship between MAC and Clinical, Biological, and Echocardiographic Parameters

We introduced parameters statistically significantly associated with MAC in a binary logistic regression model in order to investigate their combined action as risk factors for MAC. The model was achieved in order to evaluate the effects of age, diabetes, aortic atherosclerosis plaques, vascular disease, and LVEF on the probability of MAC in the investigated patients. The built model was statistically significant (*p* < 0.001) and explains 48.4% of the variation in MAC occurrence; the model’s sensibility is 88.5%, and its specificity is 64.7%. Among the five predictor variables used, two were identified as statistically significant: the presence of diabetes mellitus and the presence of vascular disease. The patients with diabetes mellitus had a 4.226 times higher risk than the others of developing MAC, and those with vascular disease had a 18.027 times higher risk than the others of developing MAC (Table 2).

### 3.5. Relationship between MAC and Thromboembolic Risk Scores

**Figure 2:**
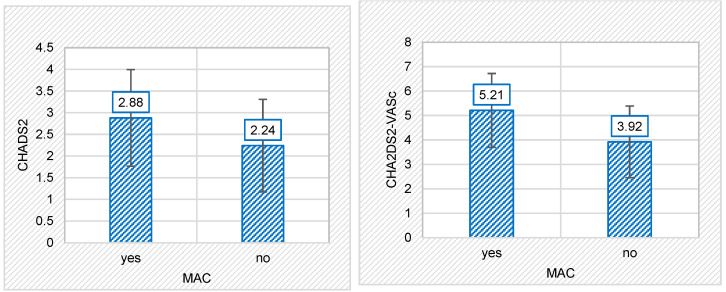
The relationship between the presence or absence of mitral annular calcification (MAC) and thromboembolic risk scores: MAC and CHADS_2_ risk score (**left**); MAC and CHA_2_DS_2_-VASc risk score (**right**).

TE risk scores were calculated for the entire patient population. Overall, the mean of the CHADS_2_ score was 2.56 ± 1.135, and for the CHA_2_DS_2_-VASc score, it was 4.57 ± 1.619. For the patients with MAC, the mean CHADS_2_ score was 2.88 ± 1.114, but in those without MAC, the mean score was 2.24 ± 1.069—a statistically significant difference (*p* = 0.005). Patients with MAC had a mean CHA_2_DS_2_-VASc score of 5.21 ± 1.513, whereas those without MAC had a mean of 3.92 ± 1.468 (again, a statistically significant difference: *p* < 0.001). The values of both TE scores were, thus, considerably higher in the MAC group as compared with the control group. The relationship between MAC and the TE risk score is shown graphically in Figure 2.

Regarding the correlations between the TE risk scores and clinical, biological, and echocardiographic parameters, it was found that the CHADS_2_ score was strongly positively correlated with age (r = 0.456, *p* < 0.001) and blood glucose values (r = 0.294, *p* = 0.003). The other biological parameters under investigation did not statistically substantially correlate with TE risk scores (Table 3).

Regarding the CHA_2_DS_2_-VASc score, it also positively and better correlated with age (r = 0.612, *p* < 0.001). Among the biological parameters, it had a weak positive correlation with blood glucose levels (r = 0.257, *p*= 0.009). No additional statistical relationships between the evaluated parameters were found (Table 3).

**Figure 3:**
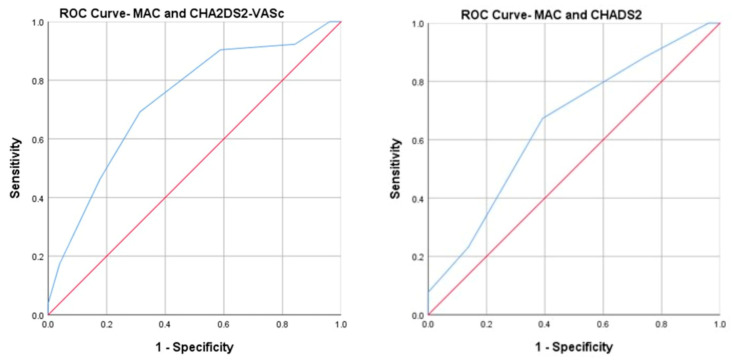
Area under the curve (ROC curve) analysis for mitral annular calcification (MAC) with CHA_2_DS_2_-VASC risk score (**left**) and with CHADS_2_ risk score (**right**).

Furthermore, ROC curve analyses (Figure 3) showed that the areas under the curve (AUCs) for CHA2DS2-VASC with MAC and CHADS2 with MAC were 0.73 (95% CI, 0.63–0.82) and 0.65 (95% CI, 0.54–0.75), respectively.

### 3.6. Relationship between MAC and Thromboembolic Risk Depending on Gender

**Figure 4:**
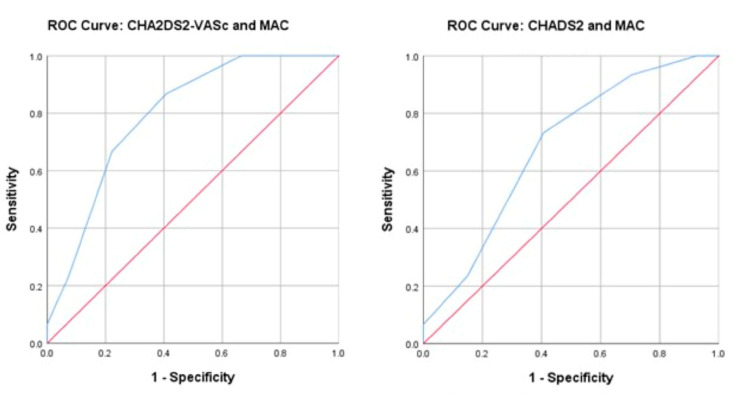
Area under the curve (ROC curve) analysis for mitral annular calcification (MAC) with CHA_2_DS_2_-VASc risk score (**left**) and CHADS_2_ risk score (**right**) in women.

Additionally, a difference in the CHADS2 score based on gender can be seen for the group with MAC. The mean for the female gender was found to be greater than for the male gender (3 ± 1.050 vs. 2.73 ± 1.203). Also, the CHA_2_DS_2_-VASc score was higher in women than in men (5.87 ± 1.167 vs. 4.32 ± 1.492). A ROC curve analysis showed that both scores were higher in women: AUC in women: 0.795 (95%CI, 0.677–0.913) for CHA_2_DS_2_-VASC and 0.683 (95% CI, 0.543–0.824) for CHADS_2_ (Figure 4).

## 4. Discussion

Mitral valve abnormalities are relatively common in the general population, and their incidence increases in patients with cardiovascular diseases, diabetes, and metabolic syndrome. We chose MAC to determine if it influences patients’ TE risk and other clinical and biological parameters, such as the association between MAC and related diseases. There are currently limited data available in the literature.

Considering that the link between MAC and TE risk is already emphasized in the most recent literature, we believe it is worthwhile to investigate the potential of including this variable in the current risk scores in order to increase their predictive power. We draw attention to the fact that, in the current AF guidelines, in the assessment of the CHA_2_DS_2_-VASc risk score, vascular disease (the letter V) is a parameter, but it strictly refers to peripheral arterial disease, myocardial infarction, and aortic atherosclerosis. Therefore, we believe that verifying the association between MAC and the TE risk score is not redundant but, on the contrary, could improve its predictive power.

This study examined the relationship between MAC and the two TE risk scores, CHADS2 and CHA2DS2-VASc, biochemical and echocardiographic differences in the studied groups, and the prevalence of other diseases.

### 4.1. Clinical Data

Old age, obesity, and a history of diabetes were the factors that mainly characterized the studied population. Congestive heart failure, prior stroke, and vascular diseases were also present in our patients. Our results were consistent with the information already available in the literature. Kanjanauthai et al. in the MESA study observed an increased incidence of MAC in elderly patients [12]. This association can be explained by the fact that MAC is considered a manifestation of atherosclerosis. Furthermore, in our study, MAC was diagnosed in a higher proportion in women than in men. Similar investigations have demonstrated the relationship between MAC and the feminine gender. This suggests a unique pathophysiological process involving calcium metabolism [13]. MAC in elderly women can be explained by postmenopausal osteoporosis and ectopic calcium deposition [14]. In cases such as diabetes, chronic kidney disease, and atherosclerosis or because of advanced age, the balance between the factors that inhibit and those that promote calcification becomes unstable, and thus, calcifications can appear at any level [15]. MAC is considered a manifestation of atherosclerotic disease since the risk factors and pathophysiological mechanisms are common [5,16].

Additionally, MAC was identified in our study as a risk factor for vascular disease. The association between MAC and carotid and peripheral arterial disease has been demonstrated before. In multiple studies, the presence of MAC was associated with a higher incidence of PAD [17,18]. For instance, in a trial that aimed to establish a connection between MAC and PAD, the mean ankle/brachial systolic pressure index was considerably lower in the MAC group than in the control group [19].

Similarly, in a study that included 17,735 patients under the age of 65 years, patients with MAC had a higher prevalence of severe IHD (including left main coronary artery and triple vessel disease) than patients without MAC. Thus, MAC represents a predictive factor for IHD, and the idea of MAC being a risk factor for vascular disease is further strengthened [20].

Our research shows that the MAC patients had a higher incidence of DM than the control group. It should be noted that, like vascular disease, this pathology is a component of the CHA_2_DS_2_-VASc risk score. A recent study on 138 diabetic individuals found a higher frequency of MAC in the population under consideration [21]. In patients with DM, the occurrence of MAC, as a form of atherosclerotic disease, can be explained by the generation of oxidative stress influenced by high blood sugar. Endothelial dysfunction occurs as a result of this process, and along with hypercholesterolemia, atherosclerosis will later develop [22]. More than that, to support this theory, blood tests of our patients with MAC showed higher blood glucose levels when compared with the control group. There is evidence that oxidative stress influences stages of certain thrombotic processes, including the activation of platelets by decreasing the bioavailability of nitric oxide [23]. Therefore, in individuals with MAC, increased oxidative stress might increase morbidity and mortality rates through TE episodes.

### 4.2. Echocardiographic Data

In our study, echocardiographic examinations revealed 52 patients with MAC. Furthermore, sinus rhythm patients with MAC showed significantly decreased LVEF values compared with those without MAC. These findings are in agreement with those of previous studies. Rao et al. demonstrated that patients with chronic kidney disease and severe MAC are associated with a decrease in LV systolic function [24]. In addition, reduced LV systolic function has been observed in diabetic patients with MAC [25]. A speckle-tracking analysis performed on 91 patients with MAC and a control group of 48 patients demonstrated that the presence of MAC is related to a decrease in LV systolic and diastolic function. Also, these changes are correlated with the severity of calcification [26]. Various mechanisms can be used to explain this observation. First of all, it is assumed that myocardial ischemia present in the small coronary vessels is involved [27]. Another argument highlighted in the literature involves both the metabolism of vitamin D and parathormone or the metabolism of calcium and phosphorus. Imbalances at this level promote the calcification of the mitral valve and, thus, LV dysfunction [27]. Lastly, systemic inflammation is associated with the presence of MAC and systolic LV dysfunction [26,28]. This link between MAC and LV systolic dysfunction may be an explanation of the cardiovascular events associated with MAC. Early diagnosis of LV systolic dysfunction is of major importance, and the unhesitant initiation of treatment can delay or prevent the onset of heart failure. Thus, in our study, the difference in LVEF values between MAC patients and the control was not significant in AF patients.

### 4.3. Thromboembolic Risk Scores

Cerebrovascular events have been associated with MAC since 1946, when Rytand et al. described the case of a patient with MAC and stroke [29]. Subsequently, Benjamin et al. demonstrated that the risk of stroke is doubled by the presence of MAC, after adjusting for other risk factors [30]. Important studies, such as the Framingham Heart Study [30], the LIFE study [6], and the Strong Heart Study [7], found similar results, headlining the increased risk of stroke in populations with MAC. Patients diagnosed with MAC have an increased risk of developing arrhythmias, for example, AF, and, of course, an increased risk of TE [31,32,33]. Now, new data from the literature reveal this increased risk even among patients without AF [5].

MAC is an independent risk factor for TE events and stroke, beyond AF or other common cardiovascular risk factors. A recent study highlighted the link between MAC and the increased risk of cerebral embolism. This study was conducted for a period of 15 years and included 6814 patients (of which 644 had MAC), and of the total number of strokes reported, 79% were ischemic strokes. Calicchio et al. observed that MAC was associated with an increased risk of all strokes after adjusting for all parameters assessed in their study [34].

Several theories have tried to explain the association between TE risk and MAC. On the one hand, some authors blame cerebral embolic events on the relationship between MAC and carotid stenosis. On the other hand, autopsies performed have described calcium emboli in the cerebral arteries of MAC patients [16]. Another theory is the relationship between MAC and atrial dysfunction or atrial fibrosis, which can cause the formation of thrombi in the left atrium [32,35]. However, some authors state that, for the time being, it cannot be established with accuracy whether TE risk is caused by calcification itself or by its association with other cerebrovascular risk factors [16].

The association between MAC and TE risk scores, like CHADS_2_ and CHA_2_DS_2_-VASc, is not well established in the literature. In our study, the average TE risk scores (CHADS_2_ and CHA_2_DS_2_-VASc) were higher in patients in the MAC group than in the control group. This finding is consistent with other recent studies that discussed this association [5,21]. The difference that we noticed between the two categories allows us to say that MAC influences both TE risk scores. Regarding the correlations between TE risk scores and clinical, biological, and echocardiographic parameters, it was observed that the CHADS_2_ score was positively correlated with blood glucose values, but with the other parameters, it had no statistical significance. The CHA_2_DS_2_-VASc score correlated positively with age, and there was an association with blood sugar levels.

This study brings to the foreground the importance of a routine clinical and paraclinical examination of patients at risk of cardiovascular events and that MAC discovered incidentally in these patients raises a red flag regarding their increased risk of TE, even in the absence of AF or other risk factors. Simple TE risk scores that are easy to use and available at any time, such as CHADS_2_ and CHA_2_DS_2_-VASc, can indicate to the clinician the need for TE prevention, increasing patient survival and life expectancy. Although its predecessor, the CHADS_2_ score, has maintained its predictive usefulness over time, it is important to remember that the CHA_2_DS_2_-VASc score is the current guideline recommendation since the greatest amount of evidence suggests that it can predict thromboembolic risk [8].

More than that, this study brings, as a novelty, the possibility of including MAC in the calculation of the CHA_2_DS_2_-VASc TE risk score, together with the other parameters of vascular disease, in order to increase its predictive value.

Since the data in the literature are limited at the moment, the current study shows that MAC seems to have multiple associations with patient mortality. Moreover, the findings of our study provide clues regarding the potential for further investigation.

### 4.4. Study Limitations

Our clinical small study was a pilot study, which provides the opportunity for future research on a larger scale with more patients and more monitored parameters over a longer period. For a more detailed description of MAC regarding its extent and location, computer tomography is the preferred method. However, the availability of this imaging method is limited and exposes the patient to irradiation. Therefore, we chose to use a less expensive, more accessible technique for early MAC detection. MAC is classified in the literature according to the extent of focal calcifications. However, this classification is not commonly applied in current practice, in which the examination is performed using echocardiography. In this study, MAC was not graded by severity. The data interpretation and results can only be applied to patients referred to a hospital for a first evaluation; they cannot be extended to the MAC population in general.

## 5. Conclusions

MAC is a progressive, chronic, age-related process, usually asymptomatic, that evolves toward important cardiovascular complications, influencing the risk of cardiovascular events and mortality. Our study reinforces the fact that simple and widely available tools such as blood tests, risk scores, and echocardiography are important in the clinical approach to patients with a high risk of MAC and TE events. We demonstrated that the CHA_2_DA_2_-VASc and CHADS_2_ scores were significantly higher in patients with MAC. Both risk scores correlated very well with the presence of MAC, but the current recommended score (CHA_2_DS_2_-VASc) had a better degree of correlation and higher statistical significance. This research highlights the possibility of incorporating MAC in the CHA_2_DS_2_-VASc risk score calculation, together with the other vascular disease indicators, in terms of enhancing its predictive value.

## Figures and Tables

**Table 1:** Demographic, clinical, laboratory, and echocardiographic characteristics of all patients included in the study (with and without mitral annular calcification).

Parameter	Overall (n = 103)	with MAC (n = 52)	without MAC (n = 51)	*p*-Value
Clinical parameters				
Age (years)	72.59 ± 9.914	74.46 ± 10.019	70.69 ± 9.528	0.042 *
BMI (kg/m^2^)	28.65 ± 5.939	29.20 ± 6.463	28.10 ± 5.378	0.669
Female gender (%)	57 (55.3%)	30 (57.7%)	27 (52.9%)	0.628
Smoking (%)	45 (43.7%)	24 (46.2%)	21 (41.2%)	0.611
Comorbidities				
Arterial hypertension (%)	86 (83.5%)	46 (88.5%)	40 (78,4%)	0.170
Diabetes mellitus (%)	31 (30.1%)	22 (42.3%)	9 (17.6%)	0.006 *
Obesity (%)	35 (34.0%)	20 (38.5%)	15 (29.4%)	0.332
Heart failure (%)	82 (79.6%)	41 (78.8%)	41 (80.4%)	0.846
Myocardial infarction (%)	7 (6.8%)	5 (9.6%)	2 (3.9%)	0.437
Prior stroke (%)	8 (7.8%)	6 (11.5%)	2 (3.9%)	0.269
Atrial fibrillation (%)	42 (40.8%)	24 (46.2%)	18 (35.3%)	0.262
Aortic atherosclerosis plaques (%)	68 (66.0%)	47 (90.4%)	21 (41.2%)	<0.001 *
Vascular disease (%)	67 (65.0%)	48 (92.3%)	19 (37.3%)	<0.001 *
Peripheral artery disease (%)	22 (21.4%)	12 (23.1%)	10 (19.6%)	0.668
Biological parameters				
Total cholesterol (mg/dL)	191.86 ± 54.270	189.00 ± 51.406	194.78 ± 57.407	0.591
Triglycerides (mg/dL)	117.75 ± 53.757	114.94 ± 59.352	120.61 ± 47.808	0.288
Fasting Glycemia (mg/dL)	120.44 ± 47.576	126.85 ± 61.046	113.78 ± 26.418	0.870
AST (U/L)	29.31 ± 39.397	32.33 ± 48.947	26.24 ± 26.522	0.484
ALT (U/L)	30.54 ± 35.991	33.13 ± 41.888	27.90 ± 28.959	0.702
GGT (U/L)	66.85 ± 122.845	94.72 ± 173.207	42.39 ± 32.441	0.633
Echocardiographic parameters				
LVEF (%)	49.50 ± 13.085	46.48 ± 13.126	52.59 ± 12.423	0.010 *
IVS (mm)	12.14 ± 1.754	12.27 ± 1.868	12.00 ± 1.637	0.263
LVPW (mm)	12.16 ± 1.685	12.25 ± 1.823	12.08 ± 1.545	0.583
LVEDD (mm)	50.82 ± 9.045	50.52 ± 8.012	51.14 ± 10.062	0.653
TE risk scores				
CHADS_2_	2.56 ± 1.135	2.88 ± 1.114	2.24 ± 1.069	0.005 *
CHA_2_-DS_2_-VASc	4.57 ± 1.619	5.21 ± 1.513	3.92 ± 1.468	<0.001 *
Treatment				
Statins (%)	70	71	69	0.623
Antiplatelets (%)	57	58	57	0.779
Oral anticoagulants (%)	39	50	27	0.018 *

* Statistical significance (*p* < 0.05); AST: alanine aspartate transferase; ALT: alanine amino transferase; BMI: body mass index; CHADS_2_ and CHA_2_DS_2_VASc: thromboembolic risk scores; GGT: gamma-glutamyl transpeptidase; IVS: interventricular septum; LVEDD: left ventricular end-diastolic diameter; LVEF: left ventricular ejection fraction; LVPW: left ventricular posterior wall; MAC: mitral annular calcification.

**Table 2:** ORs for the significant risk factors (univariate and multivariate analyses).

	Univariate Analysis		Multivariate Analysis		
	*p*	OR (95% CI)	*p*	OR (95% CI)	B Coef.
Age (years)	0.042 *	-	0.200	1.037 (0.981 ÷ 1.097)	0.036
Diabetes mellitus (%)	0.006 *	3.422 (1.383 ÷ 8.469)	0.017 *	4.226 (1.293 ÷ 13.814)	1.441
Aortic atherosclerosis plaques (%)	<0.001 *	13.429 (4.572 ÷ 39.444)	0.955	1.071 (0.100 ÷ 11.430)	0.068
Vascular disease (%)	<0.001 *	20.211 (6.290 ÷ 64.944)	0.019 *	18.027 (1.618 ÷ 200.800)	2892
LVEF (%)	0.010 *	-	0.461	0.986 (0.949 ÷ 1.024)	−0.014
Constant					−4.420

* Statistical significance where *p* < 0.05; LVEF: left ventricular ejection fraction.

**Table 3:** Correlation coefficients (Pearson linear) and the linear regression equation between the TE risk scores and the clinical, biological, and echocardiographic parameters.

	CHADS2			CHA2DS2-VASC		
	r	*p*	Regression Line	r	*p*	Regression Line
Age (years)	0.456	0.000 *	y = 0.052 ∗ x − 1.229	0.612	0.000 *	y = 0.100 ∗ x − 2.683
BMI (kg/m^2^)	0.138	0.230		0.032	0.782	
Total cholesterol (mg/dL)	−0.142	0.151		−0.006	0.952	
Triglycerides (mg/dL)	0.000	0.997		0.023	0.821	
Fasting Glycemia (mg/dL)	0.294	0.003 *	y = 0.007 ∗ x + 1.762	0.257	0.009 *	y = 0.009 ∗ x + 3.533
AST (U/L)	0.122	0.221		0.076	0.443	
ALT (U/L)	0.126	0.204		0.035	0.727	
GGT (U/L)	0.057	0.591		−0.051	0.626	
LVEF (%)	−0.161	0.104		−0.139	0.161	
IVS (mm)	0.146	0.141		0.090	0.367	
LVPW (mm)	0.106	0.285		0.042	0.676	
LVEDD (mm)	0.031	0.755		−0.050	0.619	

* Statistical significance where *p* < 0.05; AST: alanine aspartate transferase; ALT: alanine amino transferase; BMI: body mass index; GGT: gamma-glutamyl transpeptidase; IVS: interventricular septum; LVEDD: left ventricular end-diastolic diameter; LVEF: left ventricular ejection fraction; LVPW: left ventricular posterior wall.

## Data Availability

No new data were created or analyzed in this study. Data sharing is not applicable to this article.
